# Microscopic endometrial perivascular epithelioid cell nodules: a case report with the earliest presentation of a uterine perivascular epithelioid cell tumor

**DOI:** 10.1186/1746-1596-7-117

**Published:** 2012-09-03

**Authors:** Chia-Lang Fang, Yun-Ho Lin, Wei-Yu Chen

**Affiliations:** 1 Department of Pathology, School of Medicine, College of Medicine, Taipei Medical University, Taipei, Taiwan; 2 Department of Pathology, Wan Fang Hospital, Taipei Medical University, Taipei, Taiwan; 3 Deparment of Pathology, Taipei Medical University Hospital, Taipei, Taiwan; 4 Department of Pathology, School of Medicine, College of Medicine, Taipei Medical University, 250 Wu-Hsing St, Taipei 11031, Taiwan

**Keywords:** Perivascular epithelioid cell, PEComa, Lymphangioleiomyomatosis, CD10, Adenomyosis, Endometriosis, Tuberous sclerosis

## Abstract

**Abstract:**

Perivascular epithelioid cell (PEC) tumors (PEComas) are a family of related mesenchymal tumors composed of PECs which co-express melanocytic and smooth muscle markers. Although their distinctive histologic, immunohistochemical, ultrastructural, and genetic features have been clearly demonstrated, their histogenesis and normal counterpart remain largely unknown. Precursor lesions of PEComas have rarely been reported. We herein describe a tuberous sclerosis patient with microscopic PEC nodules in the endometrium of adenomyosis, pelvic endometriosis, an ovarian endometriotic cyst, and the endometrium of the uterine cavity. The nodules showed a mixture of spindle-shaped and epithelioid cells concentrically arranged around small arteries. The cells exhibited uniform nuclei, light eosinophilic cytoplasm, and immunoreactivity with HMB-45 and CD10. Some nodules revealed continuity with a PEComa in the myometrium. These findings support microscopic endometrial PEC nodules possibly being precursor lesions of uterine PEComas. The wide distribution of the nodules in the pelvis may be related to the multicentricity of PEComas in tuberous sclerosis patients. Owing to the immunoreactivity with CD10, microscopic endometrial PEC nodules may be misinterpreted as endothelial stromal cells unless melanocytic markers are stained. To the best of our knowledge, this is a case with the earliest manifestation of PEC lesions occurring in the endometrium.

**Virtual Slides:**

The virtual slide(s) for this article can be found here: http://www.diagnosticpathology.diagnomx.eu/vs/9658280017862643

## Background

Neoplasms with perivascular epithelioid cell (PEC) differentiation (PEComas), as defined by the World Health Organization, are a family of mesenchymal tumors composed of histologically and immunohistochemically distinctive PECs, characteristically expressing both melanocytic and smooth muscle markers [1]. The concept of “PEC’ was first proposed by Bonetti et al. to identify a novel cell type, which exhibited an epithelioid appearance, clear-eosinophilic cytoplasm, and a perivascular distribution in renal angiomyolipoma (AML), clear-cell sugar tumor (CCST) of the lung, and lymphangioleiomyomatosis (LAM) [2]. Since that time, PEComas have been reported at a wide variety of anatomic sites [3-5]. In the female genital tract, PEComas most frequently affect the uterus [6]. AML, LAM, sclerosing PEComa, and PEComa-not otherwise specified (NOS) were described in the uterus [6-16]. We herein report a unique case of microscopic endometrial PEC nodules, which have never been reported in the literature, and they perhaps represent the earliest manifestation of uterine PEComas. We discuss their histologic and immunohistochemical features, the relationship between the microscopic PEC nodules and PEComas of the female genital tract, and their clinical significance.

## Case presentation

### Clinical summary

A 29-year-old woman had suffered from abnormal vaginal bleeding and severe dysmenorrhea for a long time. Urinary frequency was noted, with exacerbation during menstruation. The patient was known to have tuberous sclerosis which presented with epilepsy and adenoma sebaceum when she was 19 years old. She underwent a left nephrectomy for renal cell carcinoma and a right partial nephrectomy for AML at 20 years old. Ultrasonography showed a poorly demarcated heterogeneous area measuring 4.7 cm in the largest dimension in the myometrium. Debulking surgery for adenomyosis, ablation of the pelvic endometriosis, and cystectomy of the left ovary were performed. Two years later, the patient still complained of dysmenorrhea and diarrhea aggravated during menstruation. She received a second surgery with a clinical diagnosis of endometriosis. The gynecologist found severe fibrous adhesion and endometriosis in the pelvic cavity. A supracervical hysterectomy and bilateral salpingo-oophorectomy were performed. The follow-up information revealed no evidence of recurrent or metastatic disease 168 months after the hysterectomy.

## Materials and methods

Specimens of the first surgery included two myometrial fragments, one piece of pelvic soft tissue, and some ovarian fragments. Specimens of the second surgery included the uterine corpus, bilateral ovaries, and bilateral fallopian tubes. All specimens were fixed in 10 % formalin and processed for a histologic examination by conventional methods. Hematoxylin and eosin (H&E)-stained sections were analyzed. Immunohistochemistry was performed using a Benchmark XT automated slide stainer (Ventana Medical Systems, Tucson, AZ, USA). The primary antibodies are listed in Table 1.

**Table 1:** Antibodies used in this study

**Antibody**	**Clone**	**Dilution**	**Source**
Melanosome	HMB45	1: 50	Dako, Carpentaria, CA, USA
Smooth muscle actin	1A4	1: 50	Dako, Carpentaria, CA, USA
Desmin	D33	1: 200	Dako, Carpentaria, CA, USA
CD10	56C6	1: 80	Novocastra, Newcastle Upon Tyne, UK
Progesterone receptor	16	1: 200	Novocastra, Newcastle Upon Tyne, UK
Estrogen receptor	6 F11	1: 200	Novocastra, Newcastle Upon Tyne, UK

### Pathologic findings

**Figure 1:**
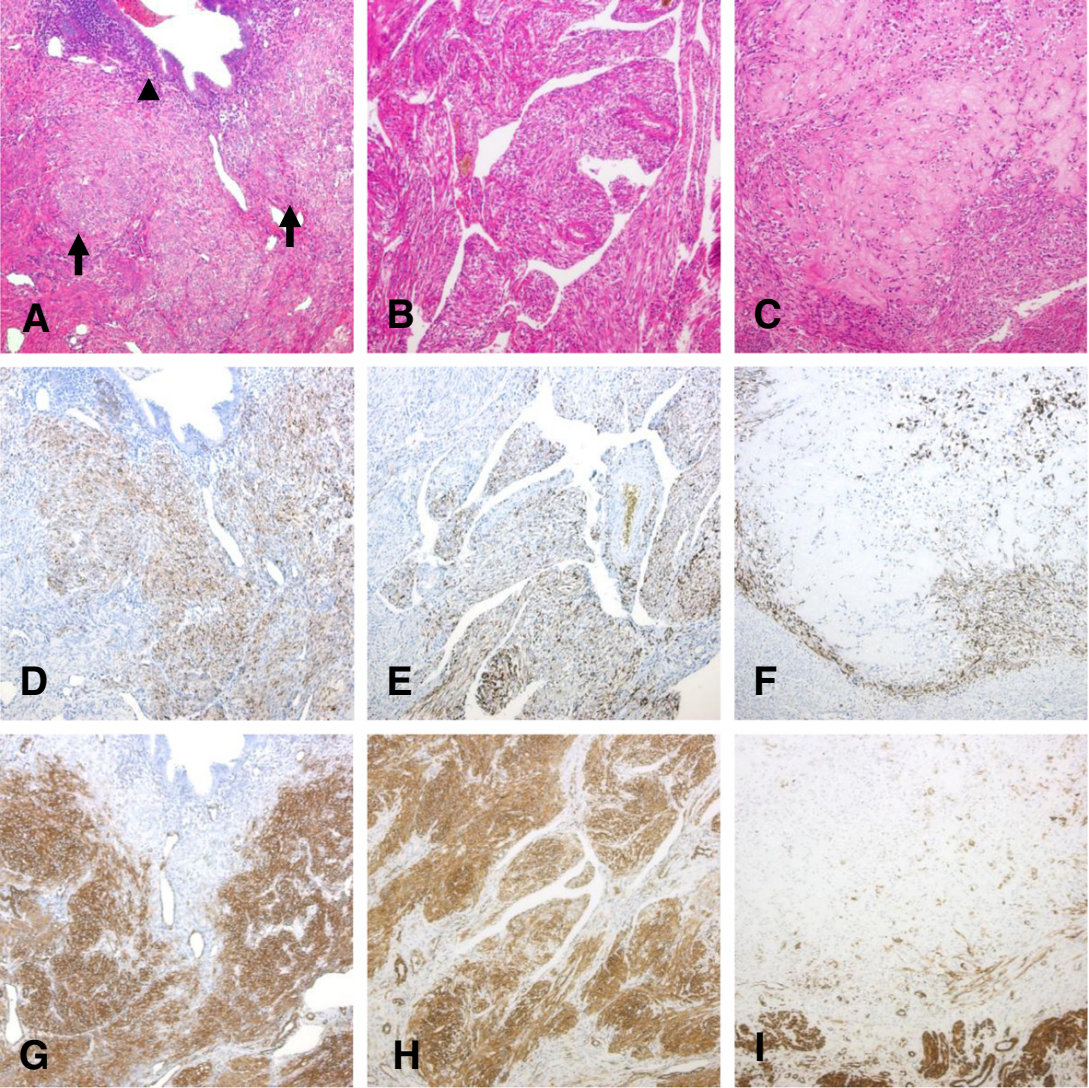
**Adenomyosis-associated perivascular epithelioid cell tumor (PEComa), lymphangioleiomyomatosis, and sclerosing PEComa (x100).** (**A**) An adenomyosis-associated PEComa with ill-defined tumor borders and light eosinophilic tumor cells (arrow). The PEComa was mainly distributed around the adenomyosis (arrowhead). (**D**) and (**G**) respectively show that the PEComa was focally positive for HMB-45 and diffusely positive for smooth muscle actin (SMA). (**B**) Lymphangioleiomyomatosis (LAM) with proliferative spindle cell fascicles in dilated vascular spaces. (**E**) and (**H**) respectively show that LAM was focally positive for HMB-45 and diffusely positive for SMA. (**C**) A sclerosing PEComa with epithelioid tumor cells in a hyalinized extracellular matrix. (**F**) and (**I**) respectively show that the sclerosing PEComa was focally positive for HMB-45 and SMA. (**A**-**C**, hematoxylin-eosin).

On macroscopic examination, both myometrial fragments of the first surgery and the subsequently excised uterine corpus showed hypertrophic myometrium with trabecular cut surfaces. Tiny depressed hemorrhagic spots were observed. The uterine corpus measured 5.0 x 5.0 x 4.0 cm and was mildly enlarged. Fibrosis and extensive hemorrhage were observed on the serosa. One 0.8-cm myoma-like nodule with a well-demarcated margin was found within the myometrium. No thickening of the endometrium was seen. Histologically, the uterine corpus revealed adenomyosis throughout the entire myometrium, extending to the serosal surface (Figure 1A). A PEComa-NOS, composed of ill-defined spindle cell fascicles, was found to mainly be distributed around the adenomyosis foci (Figure 1A). In contrast to the deeply eosinophilic hypertrophic myometrium, the spindle cells had clear to lightly eosinophilic cytoplasm. Their nuclei were oval to spindle-shaped and uniform, with no mitotic activity. The endometrial stroma of adenomyosis was focally replaced by the PEComa. LAM, characterized by poorly defined nodular proliferation of spindle cell fascicles with many tongue-like protrusions in dilated vascular spaces, was also noted in the myometrium (Figure 1B). Tumor cells of the PEComa and LAM showed diffuse immunoreactivity with smooth muscle actin (SMA) and progesterone receptor (PR) (Figure 1D, E, G, H). HMB-45 and desmin were expressed to a lesser degree. They were negative for CD10 and estrogen receptor (ER). The myoma-like nodule was a sclerosing PEComa with uniform epithelioid cells in a hyalinized extracellular matrix (Figure 1C). It showed focal HMB-45, PR, SMA, and desmin expressions (Figure 1F, I). Neither CD10 nor ER expression was observed. Ovarian and pelvic tissues, taken during the first surgery, respectively revealed an endometriotic cyst and endometriosis.

**Figure 2:**
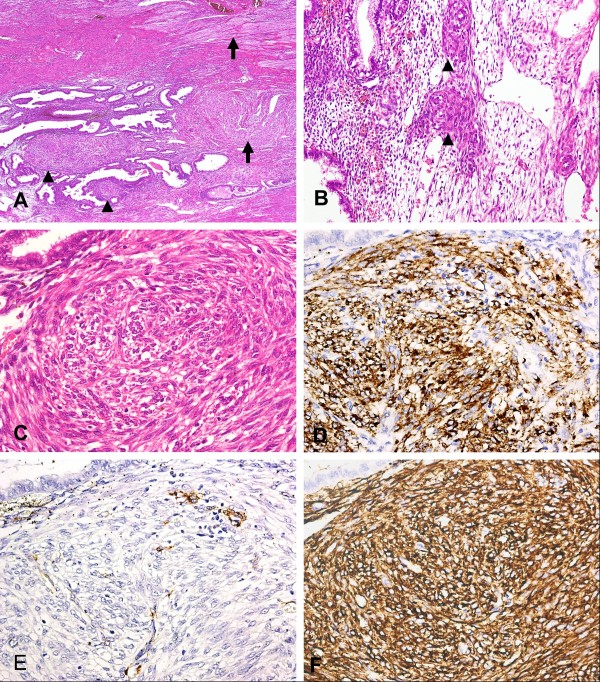
**Histologic and immunohistochemical features of microscopic perivascular epithelioid cell (PEC) nodules in the endometrium of adenomyosis.** (**A**) Microscopic PEC nodules in the endometrium of adenomyosis (arrowhead). Note the PEC tumor (PEComa) in the myometrium (arrow; x40). (**B**) and (**C**) Variably sized microscopic endometrial PEC nodules composed of spindle-shaped and epithelioid cells with a radial arrangement around small arteries (arrowhead). These nodules revealed no continuity with the PEComa in the myometrium (**B** x200, C x400). (**D**) Diffuse HMB-45 immunostaining in the microscopic endometrial PEC nodules (x400). (**E**) Rare cells with smooth muscle actin (SMA) expression in the microscopic endometrial PEC nodules (x400). (**F**) Diffuse CD10 immunostaining in the microscopic endometrial PEC nodules. Endometrial stromal cells were also positive for CD10 (x400). (A-C, hematoxylin-eosin).

**Figure 3:**
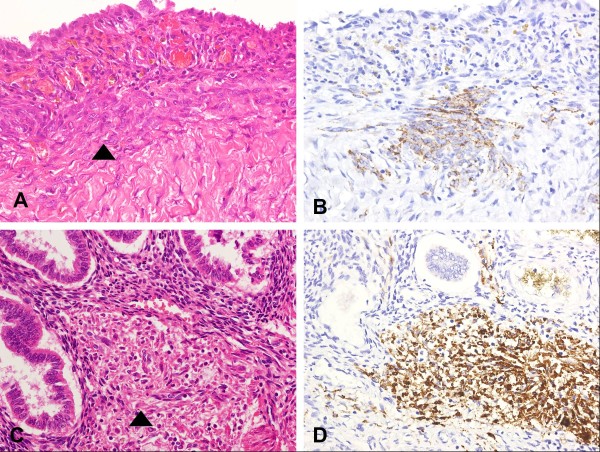
**Microscopic perivascular epithelioid cell (PEC) nodules in the endometrium of an endometriotic cyst and uterine cavity (x400).** (**A**) Microscopic PEC nodules in the endometriotic cyst (arrowhead) (hematoxylin-eosin). (**B**) HMB-45 immunostaining highlighting the microscopic PEC nodules in the endometriotic cyst. (**C**) Microscopic PEC nodules in the basal layer of the endometrium of the uterine cavity (arrowhead) (hematoxylin-eosin). (**D**) HMB-45 immunostaining highlighting the microscopic PEC nodules in the endometrium of the uterine cavity.

In addition to the PEComas and LAM, microscopic nodules composed of spindle-shaped to epithelioid cells, which were concentrically arranged around spiral arteries, were easily apparent within the ectopic endometrium of the adenomyosis (Figure 2A-C). These nodules measured < 1 mm in maximal dimension. Some of them were confined within the endometrium despite serial sections, whereas others had continuity with the PEComa in the myometrium. The spindle-shaped and epithelioid cells had indistinct cell borders, light eosinophilic cytoplasm, uniform nuclei, and no mitotic activity. They showed diffuse and strong immunoreactivity for HMB-45, CD10, and PR (Figure 2D-F). Immunoreactivity with SMA was rarely seen. Desmin and ER expressions were not detected. On the basis of the histological and immunohistochemical findings, the microscopic nodules also represented PEC lesions. Identical nodules, highlighted by HMB-45 immunostaining, were occasionally found in the ectopic endometrium of the left ovary, the pelvic endometriosis, and the basal layer of the endometrium in the uterine cavity, although they were inconspicuous on H&E-stained sections (Figure 3A-D).

## Discussion

Since HMB-45 immunoreactivity was described in renal AML and pulmonary CCST in 1991, the family of PEComas has expanded to include AML, LAM, CCST, clear-cell myomelanocytic tumor of the falciform ligament/ligamentum teres, and clear-cell tumors of other anatomic sites in the past 2 decades [1,17-21]. Although their distinctive morphologic, immunohistochemical, ultrastructural, and genetic features have been clearly demonstrated, their histogenesis and normal counterpart remain poorly understood [4]. Rare examples of early or precursor lesions of PEComas were reported in the literature [11,12,22-25]. In the current case, some microscopic PEC nodules, which were entirely confined within the endometrium with no attachment to the myometrium or the PEComa despite serial sections, were seen. These findings indicate that the nodules were not a consequence of an infiltration from the PEComa in the myometrium, supporting such nodules representing an example of early or precursor PEC lesions. We speculate that a uterine PEComa may evolve from these nodules. Proliferation of PECs initially begins around small arteries in the endometrium and produces microscopic PEC nodules. Further proliferation of PECs expands the nodule size and subsequently extends to the myometrium forming a better-developed PEComa. Only three other cases with similar early or precursor PEC lesions were reported in female genital organs, with two cases being associated with uterine PEComas and the other regarded as the earliest LAM [11,12,22]. The authors used the designation ”PEComatosis” to describe such lesions [11,12]. The clinical and pathologic features of these cases, including ours, are summarized in Table 2. The size of the early or precursor PEC lesions was usually < 2 mm. All cases, except the case reported by Liang et al., showed a similar immunohistochemical profile with diffuse positivity to melanocytic markers and focal positivity to smooth muscle markers. The case described by Liang et al. also revealed positive immunoexpressions for HMB-45 and SMA, but the extent of the staining was not described. Involvement of early PEC lesions in multiple organs was found in three patients, all of whom were complicated with tuberous sclerosis. The phenomenon may simply reflect the underlying genetic alterations associated with tuberous sclerosis, in which simultaneous or non-simultaneous development of multiple PEComas in many organs is characteristic [5]. Although patients with tuberous sclerosis are prone to having multiple PEComas and/or early PEC lesions in the female genital tract, the multicentricity of PEComas does not seem to be associated with adverse clinical outcomes [11,16]. Our patient remained free of local recurrence and metastasis 168 months after excision of the uterine corpus, ovaries, and fallopian tubes.

**Table 2:** Early or precursor perivascular epithelioid cell lesions reported in the uterus

**Reference**	**Age**	**Sites of early or precursor PEC lesions / size**	**Histopathology of early or precursor PEC lesions**	**Immunohistochemistry**	**Associated pathologic findings and important clinical features**
Fadare 2004 [11]	41	(1) Myometrium, small bowel lamina propria, and ovarian hilum.	Aggregates of epithelioid cells with eosinophilic cytoplasm and vacuolated cytoplasm in an occasional perivascular distribution, no cytologic atypia.	Positive for HMB-45, Melan-A, SMA, desmin, and PR.	(1) Cervical PEComa.
		(2) < 1 mm.			(2) Associated with tuberous sclerosis.
					(3) No recurrence or metastasis at 35 months’ follow-up.
Liang 2008 [12]	59	(1) Myometrium, cervical wall, and ovarian hilum.	Bland-looking epithelioid clear cells	(1) Positive for HMB-45, Melan-A, SMA, and myogenin.	(1) Uterine malignant PEComa and LAM of pelvic lymph nodes.
		(2) 1-5 mm.		(2) Negative for desmin, ER, and PR.	(2) Associated with tuberous sclerosis;
					(3) No follow-up data.
Clay 2010 [22]	46	(1) Myometrium.	Epithelioid cells in close approximation with lymphatic-type vessels, clear to granular and eosinophilic cytoplasm.	(1) Positive for HMB-45, Mart-1, SMA, and desmin.	(1) Early LAM
		(2) <2 mm.		(2) Negative for CD10.	(2) No tuberous sclerosis.
					(3) No follow-up data.
The present case	29	(1) Endometrium of adenomyosis, pelvic endometriosis, ovarian endometriotic cyst, and the endometrium of the uterine cavity.	Aggregates of spindle-shaped and epithelioid cells in a perivascular distribution, light eosinophilic cytoplasm, no cytologic atypia	(1) Positive for HMB-45, SMA, CD10 and PR.	(1) Uterine PEComa, sclerosing PEComa, and LAM.
		(2) < 1 mm.		(2) Negative for desmin and ER	(2) Associated with tuberous sclerosis
					(3) No recurrence or metastasis at 168 months’ follow-up

AMLs, the most common member of PEComas, were reported to have similar early PEC lesions, which were designated as microharmatomas, small mesenchymal nodules, or intraglomerular lesions [4,23-25]. Such lesions were confined within glomeruli or located outside glomeruli. They contained only epithelioid cells or a mixture of epithelioid cells and adipocytes. Immunoreactivity with HMB-45 was consistently noted in epithelioid cells. Chowdhury et al., analyzing the histologic relationship between small mesenchymal nodules and AML, found that small-sized mesenchymal nodules tended to only be comprised of epithelioid cells without adipocytes or blood vessels, compared to large-sized ones which contained epithelioid cells, adipocytes, and blood vessels [24]. These findings support small mesenchymal nodules being the buds of AMLs. Precursor PEC lesions were also described in association with a urachal PEComa [26]. The precursor lesion, remote from the main urachal PEComa, was composed of capillaries lined by HMB-45-positive clear cells. A gradual transition from the precursor lesions to invasive PEComa nests was observed.

The present case is unique for the early PEC lesions being present in the endometrium, in contrast to other early PEC lesions, which were reported in the myometrium [11,12,22] (Table 2). The endometrial distribution of early PEC lesions may account for rare uterine PEComas, which present as polypoid endometrial lesions with minimal myometrial involvement [9,10]. This also strengthens the supposition that uterine PEComas are distinctive from uterine smooth muscle tumors. Uterine PEComas being a distinct clinicopathologic entity can be challenged by frequent HMB-45 expression in epithelioid and conventional leiomyosarcomas of the uterus [27-29]. The question arises as to whether uterine PEComas are a distinctive entity or whether they represent a subgroup of HMB-45-positive smooth muscle tumors [30]. Since smooth muscle tumors typically originate in the myometrium, the endometrial origin of early PEC lesions supports uterine PEComas being a distinctive tumor. The immunophenotype of early PEC lesions, with diffuse HMB-45 and rare SMA expressions, also argues against their origin from smooth muscle cells.

Another immunophenotypic feature requiring further discussion is CD10 expression. In the present case, CD10 expression was restricted to microscopic endometrial PEC nodules, whereas the well-formed PEComa, LAM, and sclerosing PEComa in the myometrium showed negative CD10 reactivity. CD10 expression was reported in 25 % of uterine PEComas which underwent CD10 staining [6]. Although CD10 reactivity is not low in uterine PEComas, the significance of CD10 expression remains to be elucidated. Herein, we try to clarify the role of CD10. First, CD10 is commonly used to distinguish endometrial stromal nodules/sarcomas from uterine smooth muscle tumors [31]. CD10, although occasionally positive in smooth muscle tumors, is relatively specific for endometrial stromal cells. It would be interesting to determine whether or not the development of uterine PEComas is related to endometrial stromal cells. In CD10-positive uterine PEComas, tumor locations were not limited to the endometrium [10,32,33]. CD10 expression was not always detected in uterine PEComas which extensively involved the endometrium [13]. On the basis of the aforementioned findings, the hypothesis that uterine PEComas are derived from endometrial stromal cells is not supported. Second, CD10 is used as a cell surface marker of mesenchymal stem cells and tumor stem cells [34]. It is of paramount importance to understand whether CD10 is a surface marker of tumor stem cells of uterine PEComas. Unfortunately, CD10 is not routinely used in diagnosing PEComas. CD10 staining was not done in the other uterine PEComas with early or precursor PEC lesions [11,12]. More cases with early or precursor PEC lesions stained with CD10 are required to answer this question.

Moreover, since uterine PEComas may present as endometrial lesions with CD10 expression, PECs may be misinterpreted as endometrial stromal cells, particularly in an endometrial curettage specimen. Plump cells with more abundant clear to light eosinophilic cytoplasm and a radial arrangement around vessels should raise suspicion of the existence of PECs. A misdiagnosis can be avoided by awareness of the existence of endometrial PEComas and assistance with immunostaining using melanocytic markers.

## Conclusion

The present case showed microscopic endometrial PEC nodules widely distributed in the endometrium of adenomyosis, pelvic endometriosis, an ovarian endometriotic cyst, and the endometrium of the uterine cavity in a tuberous sclerosis patient. The microscopic endometrial PEC nodules were clearly demonstrated to have progressed into a uterine PEComa. Further studies focusing on early HMB-45-positive PEC nodules may be helpful in clarifying the pathogenesis of PEComas. The role of CD10 in PEComas also needs to be further evaluated.

### Consent

Written informed consent was obtained from the patient for publication of this Case Report and all accompanying images. A copy of the written consent is available for review by the Editor-in-Chief of this journal.

## Abbreviations

AML, Angiomyolipoma; CCST, Clear-cell sugar tumor; LAM, Lymphangioleiomyomatosis; PEC, Perivascular epithelioid cell; PEComa, Perivascular epithelioid cell tumor.

## Competing interests

The authors declare that they have no competing interests.

## Authors’ contribution

CLF participated in drafting the manuscript and reviewing the literature. CLF, YHL, and WYC were responsible for making the pathologic diagnosis. WYC proposed the idea and revised the manuscript. All authors have read and approved the final manuscript.

## References

[B1] Folpe AL Fletcher CDM, Unni KK, Mertens F Neoplasms with perivascular epithelioid cell differentiation (PEComas) World Health Organization Classification of Tumors. Pathology and genetics of tumors of soft tissue and bone 2002 Lyon: ARC Press 221 222

[B2] Bonetti F Pea M Martignoni G Zamboni G PEC and sugar Am J Surg Pathol 1992 16 307 308 10.1097/00000478-199203000-00013 1599021

[B3] Hornick JL Fletcher CD PEComa: what do we know so far ? Histopathology 2006 48 75 82 10.1111/j.1365-2559.2005.02316.x 16359539

[B4] Martignoni G Pea M Reghellin D Zamboni G Bonetti F PEComas: the past, the present and the future Virchows Arch 2008 452 119 132 10.1007/s00428-007-0509-1 18080139 PMC2234444

[B5] Folpe AL Kwiatkowski DJ Perivascular epithelioid cell neoplasms: pathology and pathogenesis Hum Pathol 2010 41 1 15 10.1016/j.humpath.2009.05.011 19604538

[B6] Fadare O Perivascular epithelioid cell tumor (PEComa) of the uterus: an outcome-based clinicopathologic analysis of 41 reported cases Adv Anat Pathol 2008 15 63 75 10.1097/PAP.0b013e31816613b0 18418088

[B7] Sieinski W Lipomatous neometaplasia of the uterus. Report of 11 cases with discussion of histogenesis and pathogenesis Int J Gynecol Pathol 1989 8 357 363 10.1097/00004347-198912000-00007 2807714

[B8] Gyure KA Hart WR Kennedy AW Lymphangiomyomatosis of the uterus associated with tuberous sclerosis and malignant neoplasia of the female genital tract: a report of two cases Int J Gynecol Pathol 1995 14 344 351 10.1097/00004347-199510000-00010 8598338

[B9] Pea M Martignoni G Zamboni G Bonetti F Perivascular epithelioid cell Am J Surg Pathol 1996 20 1149 1153 10.1097/00000478-199609000-00012 8764751

[B10] Vang R Kempson RL Perivascular epithelioid cell tumor (‘PEComa’) of the uterus: a subset of HMB-45-positive epithelioid mesenchymal neoplasms with an uncertain relationship to pure smooth muscle tumors Am J Surg Pathol 2002 26 1 13 10.1097/00000478-200201000-00001 11756764

[B11] Fadare O Parkash V Yilmaz Y Mariappan MR Ma L Hileeto D Qumsiyeh MB Hui P Perivascular epithelioid cell tumor (PEComa) of the uterine cervix associated with intraabdominal ‘PEComatosis’: A clinicopathological study with comparative genomic hybridization analysis World J Surg Oncol 2004 2 35 10.1186/1477-7819-2-35 15494070 PMC527874

[B12] Liang SX Pearl M Liu J Hwang S Tornos C "Malignant" uterine perivascular epithelioid cell tumor, pelvic lymph node lymphangioleiomyomatosis, and gynecological pecomatosis in a patient with tuberous sclerosis: a case report and review of the literature Int J Gynecol Pathol 2008 27 86 90 10.1097/pgp.0b013e318150df37 18156981

[B13] Froio E Piana S Cavazza A Valli R Abrate M Gardini G Multifocal PEComa (PEComatosis) of the female genital tract associated with endometriosis, diffuse adenomyosis, and endometrial atypical hyperplasia Int J Surg Pathol 2008 16 443 446 10.1177/1066896908316067 18499690

[B14] Hornick JL Fletcher CD Sclerosing PEComa: clinicopathologic analysis of a distinctive variant with a predilection for the retroperitoneum Am J Surg Pathol 2008 32 493 501 10.1097/PAS.0b013e318161dc34 18223480

[B15] Lim GS Oliva E The morphologic spectrum of uterine PEC-cell associated tumors in a patient with tuberous sclerosis Int J Gynecol Pathol 2011 30 121 128 21293289 10.1097/PGP.0b013e3181fa5a99

[B16] Yang W Li G Wei-qiang Z Multifocal PEComa (PEComatosis) of the female genital tract and pelvis: a case report and review of the literature Diag Pathol 2012 7 23 10.1186/1746-1596-7-23 PMC3378439 22404894

[B17] Pea M Bonetti F Zamboni G Martignoni G Riva M Colombari R Mombello A Bonzanini M Scarpa A Ghimenton C Fiore-Donati L Melanocyte-marker-HMB-45 is regularly expressed in angiomyolipoma of the kidney Pathology 1991 23 185 188 10.3109/00313029109063563 1664078

[B18] Pea M Bonetti F Zamboni G Martignoni G Fiore-Donati L Doglioni C Clear cell tumor and angiomyolipoma Am J Surg Pathol 1991 15 199 202 10.1097/00000478-199102000-00020 2025321

[B19] Bonetti F Pea M Martignoni G Zamboni G Iuzzolino P Cellular heterogeneity in lymphangiomyomatosis of the lung Hum Pathol 1991 22 727 728 10.1016/0046-8177(91)90298-4 2071116

[B20] Folpe AL Goodman ZD Ishak KG Paulino AF Taboada EM Meehan SA Weiss SW Clear cell myomelanocytic tumor of the falciform ligament/ligamentum teres Am J Surg Pathol 2000 24 1239 1246 10.1097/00000478-200009000-00007 10976698

[B21] Hendrickson MR Tavassoli FA Kempson RL McCluggage WG Haller U Kubik-Huch RA Tavassoli FA, Devilee P Perivascular epithelioid cell tumor World Health Organization Classification of Tumors. Pathology and genetics of tumors of the breast and female genital organs 2003 Lyon: IARC Press 243

[B22] Clay MR Gibson P Lowell J Cooper K Microscopic uterine lymphangioleiomyomatosis perivascular epithelioid cell neoplasm: a case report with the earliest manifestation of this enigmatic neoplasm Int J Gynecol Pathol 2011 30 71 75 10.1097/PGP.0b013e3181efe08d 21131829

[B23] Nagashima Y Ohaki Y Tanaka Y Misugi K Horiuchi M A case of renal angiomyolipomas associated with multiple and various hamartomatous microlesions [abtract] Virchows Arch A Pathol Anat Histopathol 1988 413 177 182 10.1007/BF00749680 3133877

[B24] Chowdhury PR Tsuda N Anami M Hayashi T Iseki M Kishikawa M Matsuya F Kanetake H Saito Y A histopathologic and immunohistochemical study of small nodules of renal angiomyolipoma: a comparison of small nodules with angiomyolipoma Mod Pathol 1996 9 1081 1088 8933519

[B25] Kiliçaslan I Güllüoglu MG Dogan O Uysal V Intraglomerular microlesions in renal angiomyolipoma Hum Pathol 2000 31 1325 1328 10.1053/hupa.2000.16533 11070127

[B26] Weinreb I Howarth D Latta E Ghazarian D Chetty R Perivascular epithelioid cell neoplasms (PEComas): four malignant cases expanding the histopathological spectrum and a description of a unique finding Virchows Arch 2007 450 463 470 10.1007/s00428-007-0378-7 17377813

[B27] Silva EG Deavers MT Bodurka DC Malpica A Uterine epithelioid leiomyosarcoma with clear cells. Reactivity with HMB-45 and the concept of PEComa Am J Surg Pathol 2004 28 244 249 10.1097/00000478-200402000-00013 15043315

[B28] Silva EG Bodurka DC Scouros MA Ayala A A uterine leiomyosarcoma that became positive for HMB45 in the metastasis Ann Diagn Pathol 2005 9 43 45 10.1053/j.anndiagpath.2004.10.011 15692950

[B29] Simpson KW Albores-Saavedra J HMB-45 reactivity in conventional uterine leiomyosarcomas Am J Surg Pathol 2007 31 95 98 10.1097/01.pas.0000213346.57391.70 17197924

[B30] Fadare O Uterine PEComa: appraisal of a controversial and increasingly reported mesenchymal neoplasm Int Semin Surg Oncol 2008 5 7 10.1186/1477-7800-5-7 18325099 PMC2278149

[B31] Chu PG Arber DA Weiss LM Chang KL Utility of CD10 in distinguishing between endometrial stromal sarcoma and uterine smooth muscle tumors: an immunohistochemical comparision of 34 cases Mod Pathol 2001 14 465 471 10.1038/modpathol.3880335 11353058

[B32] Greene LA Mount SL Schned AR Cooper K Recurrent perivascular epithelioid cell tumor of the uterus (PEComa): an immunohistochemical study and review of the literature Gynecol Oncol 2003 90 677 681 10.1016/S0090-8258(03)00325-1 13678746

[B33] Dimmler A Seitz G Hohenberger W Kirchner T Faller G Late pulmonary metastasis in uterine PEComa J Clin Pathol 2003 56 627 628 10.1136/jcp.56.8.627 12890819 PMC1770029

[B34] Maguer-Satta V Besançon R Bachelard-Cascales E Concise review: neutral endopeptidase (CD10): a multifaceted environment actor in stem cells, physiological mechanisms, and cancer Stem Cells 2011 29 389 396 10.1002/stem.592 21425402

